# *In vitro* assessment of the probiotic properties of an industrial preparation containing *Lacticaseibacillus paracasei* in the context of athlete health

**DOI:** 10.3389/fphar.2022.857987

**Published:** 2022-08-09

**Authors:** Laura Brunelli, Valerio De Vitis, Roberto Ferrari, Mario Minuzzo, Walter Fiore, Ralf Jäger, Valentina Taverniti, Simone Guglielmetti

**Affiliations:** ^1^ Sofar S.p.A., Trezzano Rosa, Italy; ^2^ Increnovo LLC, Whitefish Bay, WI, United States; ^3^ Division of Food Microbiology and Bioprocesses, Department of Food, Environmental and Nutritional Sciences, University of Milan, Milan, Italy

**Keywords:** aminoalta, CaCo-2, teer, superoxide dismutase, THP-1, NF-κB, L. paracasei DG, L. paracasei LPC-S01

## Abstract

Intense physical activity is often associated with undesirable physiological changes, including increased inflammation, transient immunodepression, increased susceptibility to infections, altered intestinal barrier integrity, and increased oxidative stress. Several trials suggested that probiotics supplementation may have beneficial effects on sport-associated gastro-intestinal and immune disorders. Recently, in a placebo-controlled human trial, the AminoAlta™ probiotic formulation (AApf) was demonstrated to increase the absorption of amino acids from pea protein, suggesting that the administration of AApf could overcome the compositional limitations of plant proteins. In this study, human cell line models were used to assess *in vitro* the potential capacity of AApf to protect from the physiological damages that an intense physical activity may cause. The obtained results revealed that the bacteria in the AApf have the ability to adhere to differentiated Caco-2 epithelial cell layer. In addition, the AApf was shown to reduce the activation of NF-κB in Caco-2 cells under inflammatory stimulation. Notably, this anti-inflammatory activity was enhanced in the presence of partially hydrolyzed plant proteins. The AApf also triggered the expression of cytokines by the THP-1 macrophage model in a dose-dependent manner. In particular, the expression of cytokines IL-1β, IL-6, and TNF-α was higher than that of the regulatory cytokine IL-10, resembling a cytokine profile characteristic of M1 phenotype, which typically intervene in counteracting bacterial and viral infections. Finally, AApf was shown to reduce transepithelial permeability and increase superoxide dismutase activity in the Caco-2 cell model. In conclusion, this study suggests that the AApf may potentially provide a spectrum of benefits useful to dampen the gastro-intestinal and immune detrimental consequences of an intense physical activity.

## Introduction

Both elite and recreational athletes commonly experience exercise-induced adverse health effects of too much exercise with the severity depending on the type and frequency of the physical activity. Strenuous exercise causes gastrointestinal symptoms in 30%–70% of athletes (Dimeo et al., 2004; Ter Steege et al., 2008; De Oliveira and Burini, 2009) and is associated with an increased risk of upper respiratory tract infections (URTIs) (Hull et al., 2012). Both adverse effects have been linked to an exercise-induced increase in intestinal permeability, altered immune function, and enhanced oxidative stress (Reid, 2001; Sadowska-Krepa et al., 2021). Several nutritional strategies have been proposed to treat or prevent the detrimental effects of physical activity, such as consuming beverages containing multiple transportable carbohydrates, limiting/avoiding fiber and solid foods hours/days before exercise, or supplementing the diet with vitamin C, D and E (Rowlands et al., 2012; Waterman and Kapur, 2012; De Oliveira et al., 2014; Cicchella et al., 2021), and antioxidants (Peake et al., 2007; Bowtell and Kelly, 2019). Supplementation with probiotics (Diaz-Jimenez et al., 2021; Tavakoly et al., 2021), “live microorganisms that, when administered in adequate amounts, confer a health benefit on the host” (Hill et al., 2014), plays an important role in maintaining normal physiology during exercise and to manage the adverse effects of those physically active. Reportedly, probiotics can positively influence human health principally by affecting the intestinal microbial ecosystem, regulating the activity of the gut-associated lymphoid tissue (GALT), and modulating the gene expression in the intestinal mucosa, so contributing to gut barrier preservation (i.e., reducing gut permeability), immune homeostasis promotion, and gut motility restoration (Dimidi et al., 2017; Ibrahim et al., 2020). These properties of probiotic microorganisms may explain the health benefits observed in athletes following probiotic supplementation, which include reduction of gastrointestinal symptoms in elite cyclists (Schreiber et al., 2021) and during a marathon race (Pugh et al., 2019), alleviation of URTI incidence after a marathon race (Tavares-Silva et al., 2021), amelioration of cardiorespiratory fitness in long-distance runners (Smarkusz-Zarzecka et al., 2020), promotion of favorable effects on self-reported muscle soreness and sleep quality in rugby players (Harnett et al., 2021), attenuation of circulating TNF-α in male baseball athletes (Townsend et al., 2018), and reduction of oxidative stress associated to exhaustive treadmill exercise in untrained subjects (Mooren et al., 2020). Moreover, recently, a probiotic formulation based on *Lacticaseibacillus paracasei* was shown to increase the maximum serum concentration of several amino acids when co-administered with plant protein (Jäger et al., 2020). The probiotic formulation used in this study, named AminoAlta™ (AApf), included 10 billion CFUs of two strains belonging to *Lacticaseibacillus paracasei*, which is one of the most commonly used bacterial species in commercial probiotic products. The species *L. paracasei* comprise numerous commercial strains including the world’s first commercial probiotic, the Shirota strain, which was demonstrated to reduce infection incidence in athletes engaged in endurance-based physical activities (Gleeson et al., 2011) and to modulate the systemic and airways immune responses post-marathon (Vaisberg et al., 2019). Hundreds of other studies, also performed with the bacterial strains of the AApf, demonstrated that *L. paracasei* can provide a plethora of benefits in a strain-specific fashion for different health conditions and through different mechanisms ranging from the modification of the bacterial community structure of the intestinal microbiota, the modulation of intestinal short-chain fatty acid levels, the regulation of evacuation frequency, and the cross-talk with the intestinal mucosal immune system (Ferrario et al., 2014; Balzaretti et al., 2015; Cremon et al., 2018; Taverniti et al., 2021).

In this study, we assessed the hypothesis that the AApf, besides improving the absorption of amino acids from plant proteins, may also exert probiotic properties that can benefit athletes’ health. To this aim, *in vitro* human cell line models have been implemented to measure the bacterial adhesion on enterocytes, the anti-inflammatory potential, the macrophage stimulatory activity, the epithelial permeability preservation, and the modulation of epithelial antioxidant capacity.

## Materials and methods

### Composition of the AminoAlta™ probiotic formulation

AminoAlta™ is a commercial probiotic product composed of 10 billion CFUs of *Lacticaseibacillus paracasei* cells [5 billion CFUs of strain *L. paracasei* LP-DG® (CNCM I-1572) and 5 billion CFUs of strain *L. paracasei* LPC-S01 (DSM 26760)] dispensed in a sachet containing 2.4 g of lyophilized product.

### Bacterial adhesion to the Caco-2 cell layer

The adhesion of AApf to a Caco-2 cell layer was assessed as previously described (Guglielmetti et al., 2008) with few changes. In brief, Caco-2 cells were grown at 37°C in an atmosphere of 95% air and 5% carbon dioxide in Dulbecco’s Modified Eagle’s Medium (MEM) supplemented with 10% (v/v) heat-inactivated fetal calf serum, 100 U/ml penicillin, 100 mg/ml streptomycin, 0.1 mM non-essential amino acids, 2 mM L-glutamine. For adhesion experiments, differentiated Caco-2 cells were used (i.e., 15 days after confluence). Approximately 2 × 10^8^ bacterial cells were incubated with a monolayer of approximately 1 × 10^6^ Caco-2 cells for 1 h at 37°C. Monolayers were washed three times with phosphate-buffered saline pH 7.3 (PBS) to release unbound bacteria and incubated with 3 ml of methanol for 8 min at room temperature to fix cells. Afterwards, cells were stained with 3 ml of Giemsa stain solution (1:20; Carlo Erba, Milano, Italy) and left 30 min at room temperature in the dark. Finally, monolayers were washed three times with PBS, dried in an incubator for 1 h, and examined microscopically (magnification, ×400) under oil immersion. All experiments were performed in triplicate.

### Study of NF-κB activation in Caco-2 cells

The activation of the nuclear factor κB (NF-κB) was studied by means of a recombinant Caco-2 cell line stably transfected with vector pNiFty2-Seap (InvivoGen, Labogen, Rho, Italy) as in Taverniti et al. (Taverniti et al., 2013). In brief, recombinant Caco-2 monolayers (approximately 5 × 10^5^ cells/well), cultivated in the presence of 50 μg/ml zeocin, were washed with PBS and then incubated with 5 × 10^7^ bacterial cells of the AApf suspended in fresh DMEM containing 100 mM HEPES (pH 7.4), resulting in a “multiplicity of infection” (MOI) of approximately 100. In a different set of experiments, Caco-2 monolayers were also co-incubated with six different commercial supplements of partially hydrolyzed vegetal proteins (PHVP; 10 μg/ml final concentration) resuspended in PBS. The tested commercial supplements were as follows: NUTRALYS pea protein (consisting of pea proteins; code PHVP-A), Organic protein™ (pea, brown rice and chia seed proteins; PHVP-B), KOS^®^ (pea, coconut milk and flax seed proteins; PHVP-C), Sunwarrior® (pea and hemp proteins; PHVP-D), Vega protein (pea and brown rice proteins; PHVP-E), Raw Organic Protein-Garden of life (pea and brown rice proteins; PHVP-F). Pro-inflammatory stimulation of Caco-2 cells was carried out by adding 2 ng/ml of interleukin (IL)-1β. After incubation at 37°C for 4 h, the activity of the secreted embryonic alkaline phosphatase (SEAP) reporter enzyme was quantified in the supernatant using the Quanti-Blue reagent (Invivogen) according to the manufacturer’s protocol using a microplate reader (Multiskan SkyHigh, Thermo Fisher Scientific, Waltham, MA) at 655 nm OD. Three independent experiments were conducted in duplicate for each condition.

### THP-1 human macrophage cell line activation assessment: Cell culture, growth conditions, and stimulation protocol

The growth medium for THP-1 cells consisted of RPMI 1640 medium (Lonza, Basel, Switzerland) supplemented with 10% (v/v) fetal bovine serum (FBS) (Gibco-BRL, Life Technologies, Milan. Italy), 2 mM L-glutamine, 100 units/ml penicillin and 100 μg/ml streptomycin (Sigma-Aldrich Pty Ltd., Darmstadt, Germany). Cells were seeded at a density of 1 × 10^6^ cells/well in 12-well plates and incubated at 37°C in a humidified atmosphere of 95% air and 5% CO_2_. Differentiation was induced by treating cells with 20 ng/ml of phorbol-12-myristate-13-acetate (PMA; Sigma-Aldrich) for 24 h. Afterwards, cells were washed once with sterile PBS to remove non-adherent cells. One hour before the bacteria were added to the cells, the culture medium was replaced with RPMI 1640 medium without FBS to allow the cells to adapt. Finally, differentiated THP-1 cells were stimulated with bacteria at MOIs 10 and 50 for 4 h.

### Preparation of RNA and real-time quantitative reverse transcription PCR (qRT-PCR)

After incubating THP-1 cells at 37°C for 4 h, the supernatant was carefully removed from each well and the total cellular RNA was isolated from adhered THP-1 cells with the RNeasy Plus Mini Kit—Qiagen (Qiagen). Afterwards, RNA concentration and purity was determined by spectrophotometric analysis (Multiskan SkyHigh, Thermo Fisher Scientific) and through electrophoresis on 0.8% agarose gel stained with GelRed Nucleic Acid Staining (Millipore). Total mRNA reverse transcription to cDNA was performed with the RNeasy Mini Kit (Qiagen) with 1 µg of RNA, using the following thermal cycle: 2 min at 42°C for DNase activity, then 15 min at 42°C, and 3 min at 95°C, as per the manufacturer’s instructions. qRT-PCR was carried out to measure the mRNA expression levels of cytokine genes using the SsoFast EvaGreen Supermix (Bio-Rad) on a Bio-Rad CFX96 system according to the manufacturer’s instructions. The primers used were as follows (5′-3′): IL-10 forward AGC​AGA​GTG​AAG​ACT​TTC​TTT​C; IL-10 reverse CAT​CTC​AGA​CAA​GGC​TTG​G; TNF-α forward TCAGCTCCACGCCATT; TNF-α reverse CCC​AGG​CAG​TCA​GAT​CAT; IL-1β forward TGG​CAA​TGA​GGA​TGA​CTT​GTT​C; IL-1β reverse CTG​TAG​TGG​TGG​TCG​GAG​ATT; IL-6 forward CGG​TAC​ATC​CTC​GAC​GGC​AT; IL-6 reverse TCA​CCA​GGC​AAG​TCT​CCT​CAT. All primers were designed previously, and their specificity was assessed with melting curves during amplification and by 1% agarose gels (Taverniti et al., 2012). Quantitative PCR was carried out according to the following thermal cycle: initial hold at 95°C for 30 s and then 41 cycles at 95°C for 10 s and 60°C for 5 s. Gene expression was normalized to the reference genes β-actin (ACTB) and Ribosomal Protein L37a (RPL37A). The primers used were as follows (5′-3′): ACTB forward ATT​GCC​GAC​AGG​ATG​CAG​AA; ACTB reverse GCT​GAT​CCA​CAT​CTG​CTG​GAA; RPL37A forward ATT​GAA​ATC​AGC​CAG​CAC​GC; RPL37A reverse AGG​AAC​CAC​AGT​GCC​AGA​TCC. The amount of template cDNA used for each sample was 25 ng. All results regarding cytokine mRNA expression levels are reported as the fold of induction (FOI) respective to the control (namely unstimulated THP-1), to which we attributed a FOI of 1.

### Assessment of the transepithelial electrical resistance (TEER) in Caco-2

The effect of the probiotic formulation on epithelial integrity was assessed with a Caco-2 cell layer by transepithelial electrical resistance (TEER) measured after 4 and 24 h. For the analysis, a fully differentiated Caco-2 cell layer obtained by growing cells in 6 well plate transwell inserts for 21 days was used. The apical side of the Caco-2 cell layers was incubated with the AApf resuspended in DMEM medium without antibiotics at a MOI of 100. TEER was measured using an epithelial voltohmeter (Milicell ERS-2 Voltohmmeter; World Precision Instruments, Hitchen, UK). This instrument uses a pair of electrodes (“chopsticks”): one electrode was placed inside the basolateral culture medium, and the shorter electrode was placed in the transwell insert within the apical culture medium. Cells were never in contact with the electrodes. Instrument calibration and the experiment were carried out according to the manufacturer’s instructions.

### Assessment of the cellular antioxidant activity in Caco-2 cells

The cellular antioxidant activity (CAA) of AminoAlta™ was evaluated with the Caco-2 cell model by measuring the relative amount of intracellular reactive oxygen species (ROS) as describe in Wolfe and Liu (Wolfe and Liu, 2007) and Xing et al. (Xing et al., 2015) with few modifications. In brief, Caco-2 cells were seeded at a density of 1 × 10^4^ cells/well on a black 96-well microplate with clear bottom in 100 μl DMEM complete medium for 72 h at 37°C to reach 80% confluence. Then, Caco-2 cells were treated with 100 μl of 10 μM 2′,7′-dichlorofluorescin diacetate (DCFH-DA, Sigma-Aldrich) up to 30 min at 37°C. Subsequently, the cells were washed with PBS and treated with 100 μl of different concentration of 2,2ʹ-azobis (2-methylpropionamidine) dihydrochloride (ABAP; from 0.2 to 2 mM), together with suspensions of AApf cells dissolved in Hanks’ balanced salt solution at multiplicity of infections (MOI) of 50, 100, 200, and 1,000. N-acetyl cysteine was used as positive control (not shown). Fluorescence was measured with Fluoroskan Ascent FL (Thermo Fisher Scientific) for 6 cycles at 5-min intervals (λ excitation = 485 nm and λ emission = 538 nm). Fluorescence measurements of Caco-2 cells treated with DCFH-DA were used as blank, whereas measurements of cells treated with DCFH-DA and ABAP (without the treatment with AApf) were used as the control. Blank was subtracted from fluorescence measurements. For CAA assessment, the higher fluorescence emissions (expressed as relative fluorescence units, RFUs) were compered between samples (i.e., AApf-treated) and controls.

### Statistical analysis

Statistical calculations were performed using the software program GraphPad Prism 5. A two-tailed unpaired Student’s t-test was used to find the significant difference between two groups. Before *t*-test, one-way analysis of variance (ANOVA) was performed for data with more than two groups. *p* value <0.05 was considered for statistical significance. All the observed significant differences had *p* < 0.05 also with a non-parametric test (Mann-Whitney U test; not shown).

## Results

### The bacteria in the AminoAlta™ probiotic formulation (AApf) possess the ability to adhere on Caco-2 enterocyte-like cells

**FIGURE 1:**
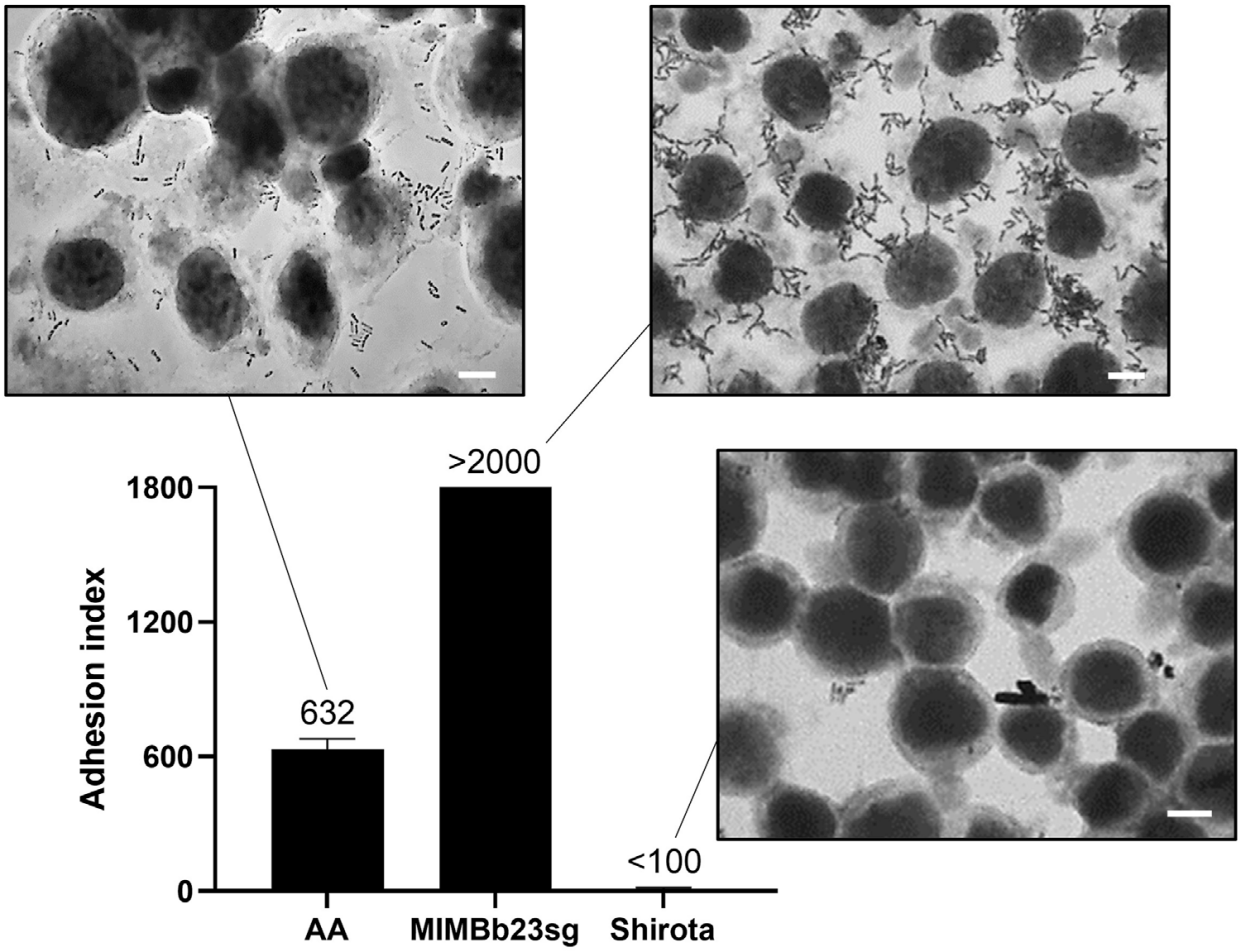
Adhesion of the bacterial cells within the AminoAlta™ probiotic formulation to a Caco-2 cell monolayer as observed with Giemsa staining under a light microscope. Adhesion quantification is reported as adhesion index (i.e., bacterial cells adhered to 100 Caco-2 cells). Histograms represent means of at least three independent experiments conducted in duplicate. The vertical bars indicate standard deviations. AA, AminoAlta™ formulation; MIMBb23sg, *Bifidobacterium bifidum* MIMBb23sg (positive control); Shirota, *Lacticaseibacillus paracasei* Shirota (negative control). White bar, 10 μm.

The differentiated Caco-2 epithelial cell layer was used to test the potential ability of the bacterial cells within the AApf to adhere on human enterocytes. The experiment was carried out including two control strains, *Bifidobacterium bifidum* MIMBb23sg and *Lacticaseibacillus paracasei* Shirota, which have been previously demonstrated to be strongly adhesive (Guglielmetti et al., 2009) and non-adhesive (Botes et al., 2008; Balzaretti et al., 2015), respectively. The reference bacteria performed as expected, with an adhesion index (i.e., bacterial cells per 100 Caco-2 cells) higher than 2000 for the *B. bifidum* strain and lower than 100 for the Shirota strain (Figure 1). Furthermore, also the bacterial cells within the AApf displayed an adhesive phenotype, corresponding to an adhesion index of about 600 (Figure 1).

### AApf prevents NF-κB activation in Caco-2 epithelial cells under inflammatory stimulation

**FIGURE 2:**
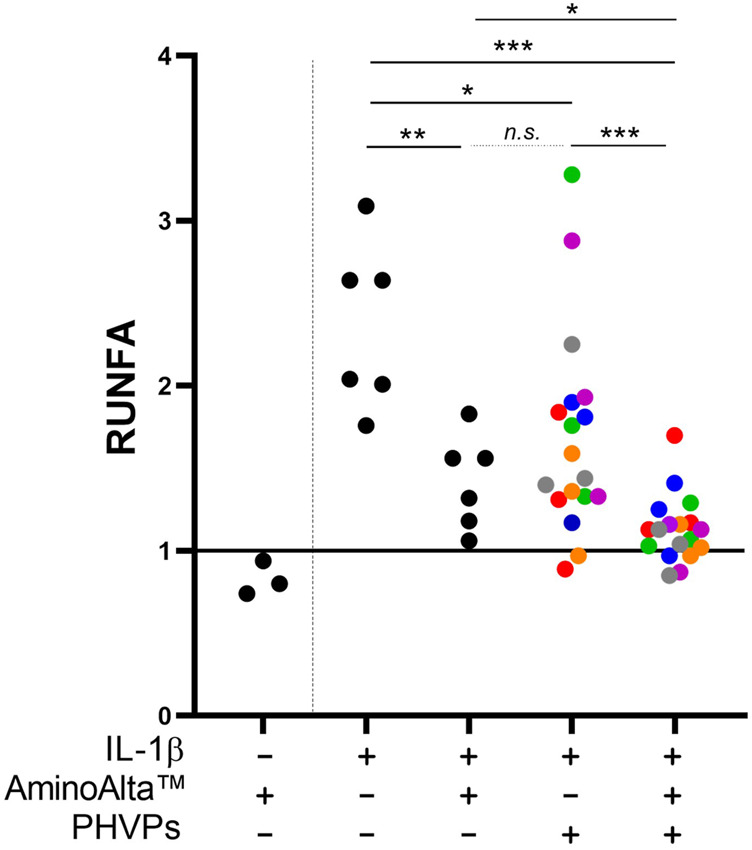
Study of the activation of the NF-κB transcriptional regulator in a Caco-2 cell layer transfected with an alkaline phosphatase (SEAP) reporter vector. Measurements of the SEAP activity were carried out after incubation of the Caco-2 cell layer without or with stimulation with IL-1β (2 ng/ml), the AminoAlta™ probiotic formulation, and different partially hydrolyzed vegetal protein commercial preparations (PHVPs). RUNFA, relative units of NF-κB activation, calculated as normalized SEAP activity. Asterisks indicate statistically significant differences according to a two-tailed unpaired Student’s t-test. ***, *p* < 0.001; **, *p* < 0.01; *, *p* < 0.05; n. s., not significant.

The anti-inflammatory activity of AApf was assessed using the Caco-2/NF-κB reporter system. The experiments were carried out by stimulating for 4 h the recombinant Caco-2 cell layer at baseline and in presence of a pro-inflammatory stimulation with IL-1β. At baseline, AApf only marginally affected NF-κB activation [relative units of NF-κB activation (RUNFA) of 0.8 ± 0.1, as mean ± standard deviation] (Figure 2). The addition of IL-1β approximately doubled the activation levels of NF-κB (RUNFA = 2.4 ± 0.5). Notably, the presence of AApf significantly reduced NF-κB activation to levels close to those at baseline (RUNFA = 1.4 ± 0.3) (Figure 2). Subsequently, the same test was carried out incubating the recombinant Caco-2 cell layer in the presence of six different commercial preparations of partially hydrolyzed vegetal proteins (PHVPs) together with the inflammatory stimulus IL-1β. NF-κB activation was slightly but significantly reduced by the PHVP formulations (RUNFA = 1.7 ± 0.6). Notably, the reduction was greater when AApf was added (RUNFA = 1.1 ± 0.2), suggesting an additive or synergistic action between AApf and PHVPs (Figure 2).

### AApf triggers the expression of cytokines by THP-1 cells

**FIGURE 3:**
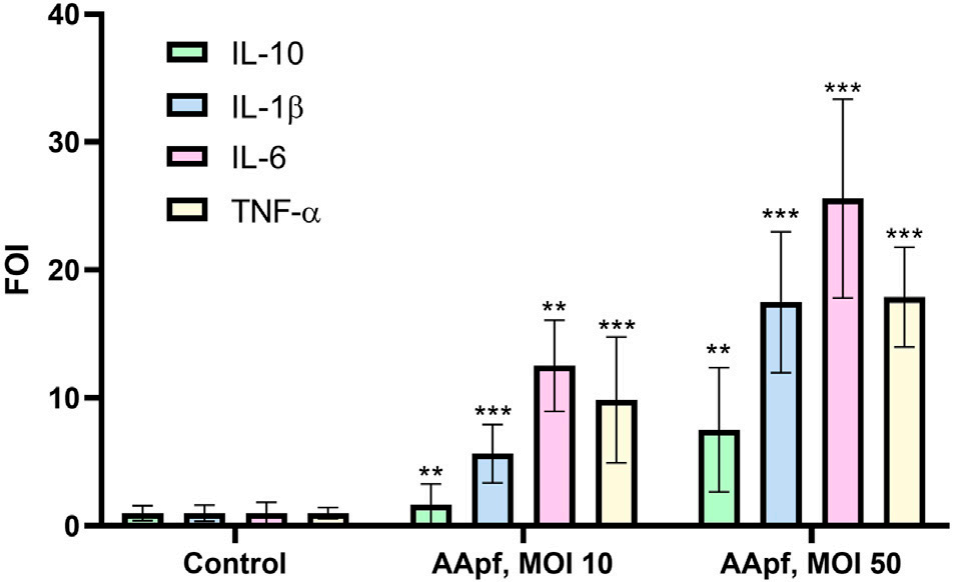
Gene expression analysis by qRT-PCR in THP-1 human macrophages unstimulated (Control) and after 4 h of stimulation with the AminoAlta™ probiotic formulation (AApf) at a multiplicity of infection (MOI) of 10 and 50. Expression levels of IL-10, IL-1β, IL-6, and TNF-α are shown as the fold change in induction (FOI) relative to expression by the control (unstimulated macrophages), which was set at a value of 1. Data are means of results from three (control and MOI 10) or two (MOI 50) independent experiments ± standard deviations. Asterisks indicate statistically significant differences (according to a two-tailed unpaired Student’s t-test) from results for unstimulated THP-1 cells. ***, *p* < 0.001; **, *p* < 0.01.

We used the THP-1 cell line as a simplified human macrophage model (Tedesco et al., 2018) to test the immunostimulatory properties of AApf at two different concentrations of bacterial cells: MOI 10 and 50. To this aim, we quantified by RT-qPCR the gene expression of tumor necrosis factor (TNF)-α, IL-1β, IL-6, and IL-10. Both AApf concentrations triggered the expression of cytokines in a dose-dependent manner (Figure 3). In particular, the AApf stimulus increased the expression of the cytokines IL-1β (FOI 5.7 ± 2.3 at MOI 10 and 17.5 ± 5.5 at MOI 50), IL-6 (FOI 12.5 ± 3.6 at MOI 10 and 25.6 ± 7.8 at MOI 50), and TNF-α (FOI 9.9 ± 4.9 at MOI 10 and 17.9 ± 3.9 at MOI 50) more than that of the regulatory/anti-inflammatory IL-10 (FOI 1.7 ± 1.6 at MOI 10 and 7.5 ± 4.9 at MOI 50) (Figure 3), suggesting that THP-1 macrophages stimulated with AApf are more prone to express markers that characterize a M1 phenotype (IL-1ß, TN-α, and IL-6) rather than a M2 phenotype (IL-10) (Chanput et al., 2013).

### AApf preserves the transepithelial permeability of the Caco-2 epithelial cell layer

**FIGURE 4:**
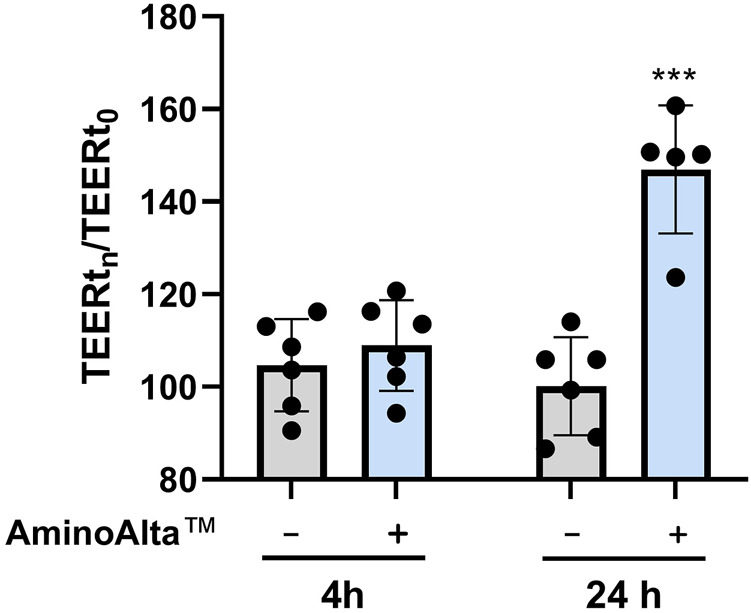
Transepithelial electrical resistance (TEER) measurements of Caco-2 cell layers exposed to the AminoAlta™ probiotic formulation (+) or unstimulated (−) after 4 and 24 h. Data are means of results from six independent experiments ± standard deviations. Asterisks indicate statistically significant differences according to a two-tailed unpaired Student’s t-test. ***, *p* < 0.001.

The ability of AApf to influence transepithelial permeability was assessed on Caco-2 monolayer as changes in the transepithelial electrical resistance (TEER) values at two incubation time points (4 and 24 h). To calculate the relative changes in TEER, the measurement was immediately performed after the stimulation of Caco-2 with AApf (TEERt_0_). Cell monolayers without bacterial incubation were considered as a control group. The obtained results showed that AApf, employed at the same bacterial cell concentration adopted for the NF-κB activation test, can significantly enhance the TEER of the Caco-2 layer after 24 h of incubation (Figure 4), suggesting its potential ability to ameliorate the epithelial barrier function.

### AApf exerts an antioxidative activity in Caco-2 cells

**FIGURE 5:**
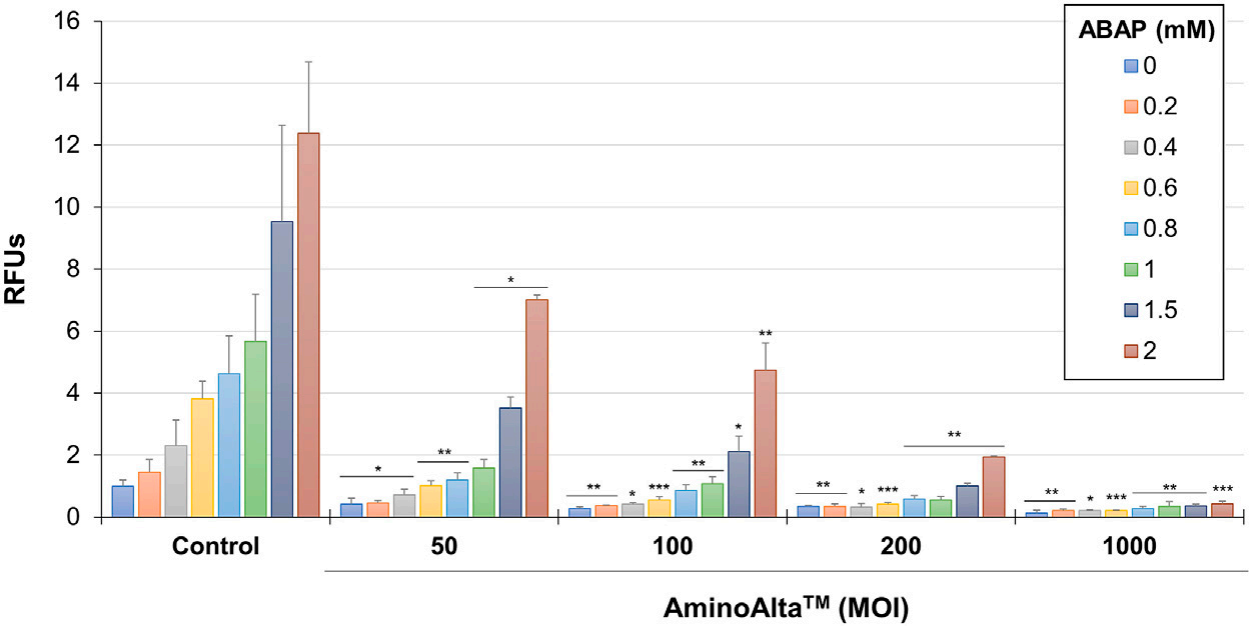
Cellular antioxidant activity of AminoAlta™ assessed in Caco-2 cells damaged by different concentrations of 2,2′-azobis (2-methylpropionamidine) dihydrochloride (ABAP). Control, Caco-2 cells without AminoAlta™ treatment. RFUs, relative fluorescence units. MOI, multiplicity of infection. Asterisks indicate statistically significant difference compared to the corresponding control as determined through a two-tailed unpaired Student’s t-test. *, *p* < 0.05; **, *p* < 0.01; ***, *p* < 0.001.

The CAA assay was used to reveal the total antioxidative capacity of the probiotic formulation under study by measuring ROS accumulation in Caco-2 cells. The experiment was carried out damaging Caco-2 cells with different concentrations of ABAP, and testing AApf at 4 different MOIs. The obtained results showed a dose-dependent ability of AApf to lower ROS in Caco-2 cells, with a significant reduction already observed at the lowest bacterial cell concentration tested (MOI = 50) (Figure 5).

## Discussion

Increasing attention is given to the use of probiotic supplements to protect from the undesirable physiological changes that may be induced by strenuous physical activity. According to the available scientific literature, the effectiveness of probiotic supplementation in athletes can is associated with improved gut-barrier-function, the attenuation of inflammatory responses, and the improvement of the epithelial antioxidant status after exhaustive exercise (Marttinen et al., 2020; Diaz-Jimenez et al., 2021). Thus, in a recent consensus document, the International Society of Sports Nutrition (ISSN) stated that certain probiotics may reduce URTIs and improve the integrity of the gut-barrier function in athletes (Jäger et al., 2019). Nonetheless, the ISSN also highlighted the importance to assess the potential properties of probiotics in trials carried out for any specific formulation, since probiotic health-promoting capabilities are strain-specific, particularly regarding immunomodulation (Hill et al., 2014); and depend on dose, delivery form, and method of administration (Jäger et al., 2019). In addition, any aspect of a probiotic formulation, including excipients and the protocol adopted in the industrial manufacturing process, may influence the properties of a probiotic product (Fiore et al., 2020). It is, in fact, demonstrated for a few well-known commercial probiotic strains that expression and presence of probiotic niche factors and effector molecules may be affected during industrial production (Duboux et al., 2021). For these reasons, in our study, we did not test microbial cells prepared in small laboratory scale (as often done in other studies), but we employed the industrial bacteria within the final commercial probiotic product.

The ability to adhere on the intestinal epithelium is conventionally considered a primary prerequisite of probiotic microorganisms, which can favor the transient colonization, pathogens exclusion, and the crosstalk with the intestinal mucosa. Thus, we first tested the ability of the bacterial cells within the AApf to adhere on the differentiated Caco-2 cell layer, which is a model for the intestinal epithelial barrier (Sambuy et al., 2005) commonly used to test the adhesion ability of probiotics (Guglielmetti et al., 2008; Guglielmetti et al., 2009). The obtained results demonstrated that the AminoAlta™ bacteria possess a significant adhesion ability, which is a feature reported to be strain-specific among members of the *Lacticaseibacillus paracasei* species (Balzaretti et al., 2015; Fonseca et al., 2021).

Subsequently, we tested the immunomodulatory properties of the AminoAlta™ formulation by assessing in epithelial Caco-2 cells the ability to reduce the activation of NF-κB, a transcriptional factor regulating the expression of proinflammatory cytokines (Baldwin, 1996) that is exploited as a therapeutic target in human inflammatory diseases (Yamamoto and Gaynor, 2001). Intense long physical exercise induces inflammation systemically and in the gut of athletes (Van Wijck et al., 2011). In our model, we used IL-1β to mimic a pro-inflammatory stimulus. The resulting increase in NF-κB activation was significantly mitigated by the presence of the AApf. This result confirms previous findings that showed that the *L. paracasei* bacteria contained in the AApf can reduce NF-κB activation in the same *in vitro* cell line under inflammatory stimulation (Balzaretti et al., 2015). Here, we additionally demonstrated that AminoAlta™ bacteria exert anti-inflammatory activity also when employed as industrial lyophilized biomass within the 4 h of experiment time.

The anti-inflammatory properties of *L. paracasei* were proposed to be connected to an unidentified peptide factor produced by the bacterium during the fermentation process (Zagato et al., 2014; Balzaretti et al., 2015), which can potentially derive from the hydrolysis of the proteins/polypeptides present in the culture medium. Interestingly, we found an increase of the anti-inflammatory effect when the AApf was used in the experiment together with commercial preparations of partially hydrolyzed plant proteins (PHVPs). Animal and vegetable protein hydrolysates have been shown to exert anti-inflammatory properties in several studies (Kiewiet et al., 2018; Kim et al., 2021; Montserrat-De La Paz et al., 2021). Also in our experiments, the different PHVPs exerted a mild but significant reduction of NF-κB activation in Caco-2 cells, which was significantly enhanced by the presence of the AApf. The AminoAlta™ bacteria were shown in a previous publication to be able to hydrolyze plant (rice and pea) proteins (Jäger et al., 2020). Therefore, we can hypothesize that the bacteria in the AApf carried out a partial additional hydrolysis of the vegetal proteins during the 4 h of co-incubation in contact with Caco-2 cells, releasing peptides that may potentially exert anti-inflammatory activities. Reportedly, the ability of lactic acid bacteria to generate anti-inflammatory peptides from the hydrolysis of food proteins has been already reported for instance for casein (Stuknyte et al., 2011) and cereal proteins (Galli et al., 2018).

Intense and prolonged (arduous) exercise is reported to provoke immunity suppression and increased susceptibility to infections (Simpson et al., 2020). Strenuous physical activity, in fact, has the potential to alter transiently (i.e., from hours to days) immune functionality, including NK cell activity, and antigen presentation and cytokine production by monocytes/macrophages (Woods et al., 2000; Nieman and Wentz, 2019). In our study, the AApf was shown to trigger cytokine gene transcription in macrophages *in vitro*. Macrophages are immune cells that sense the molecules and stimuli present in the microenvironment and polarize towards a specific phenotype depending on the surrounding conditions, with a high level of plasticity between the M1 and M2 phenotypes that, once activated, can permit the resolution of inflammation (Liu et al., 2020). Specifically, the observed increased expression of pro-inflammatory cytokines IL-1β, IL-6, and TNF-α evokes a pattern of markers typical of the M1 phenotype. This population of activated macrophages is associated to increased phagocytic capacity, tumor regression and defense against bacterial and viral infection (Ley, 2017). In particular, M1 polarized macrophages are the first line of defense against intracellular pathogens through mechanisms of endocytosis, production of reactive oxygen species (ROS), increase of antigen-presenting ability, and also by the induction of a Th1 switch on CD4 T cells, which in turn potentiate the response against infections (Atri et al., 2018).

Our results show that AApf may exert both anti-inflammatory (reduced activation of NF-κB) and pro-inflammatory (enhanced expression of cytokines) activities. However, these results are not in contradiction, since they were obtained in different cell models. The NF-κB activation was assessed in Caco-2 cells, i.e. an enterocyte model, whereas the induction of pro-inflammatory cytokines in THP-1 cells, which are monocytes differentiated to macrophages. The intestinal epithelial cells are continuously exposed to different microbial cells and, for this reason, they trained to mainly develop tolerogenic responses. On the contrary, macrophages are antigen presenting cells that work as sentinels of the immune system, in charge of patrolling the environment, being more prone to rapidly mount inflammatory responses upon the (first) encounter with microbial cells. The different immunomodulatory attitude in the cross-talk towards epithelial or proper immune cells have previously been reported for probiotic bacteria. For instance, *Lacticaseibacillus paracasei* strain DG displayed an anti-inflammatory phenotype on epithelial cells, by reducing NF-κB in Caco-2 model (Balzaretti et al., 2015), whereas showed an immunostimulatory phenotype when used to stimulate THP-1 cells, by inducing IL-8, TNF-α and CCL20 (Balzaretti et al., 2017). Analogously *Lactobacillus helveticus* MIMLh5 was shown to strongly reduce NF-κB activation in Caco-2 cell layer, while elicited a Th1 response in U937 macrophage cell line and in macrophages isolated from mouse bone marrow (Taverniti et al., 2013).

Strenuous exercise was also associated with the disruption of the intestinal barrier integrity, resulting in increased gut permeability (Janssenduijghuijsen et al., 2016; Ribeiro et al., 2021), causing local and systemic low-grade chronic inflammation, which is mechanistically linked with several pathological conditions ranging from inflammatory bowel diseases and metabolic syndrome to food allergy and celiac disease (Bischoff et al., 2014). Probiotic administration has been often proposed as a useful tool to improve intestinal barrier function, nonetheless convincing experimental data are still missing, and a wide variation exists among different microbial strains (Ramos et al., 2013; Bron et al., 2017). Reportedly, the probiotic mixture VSL#3 was shown to protect the epithelia barrier by preserving tight junction protein expression in a mouse model of colitis (Mennigen et al., 2009) and in a rat model of alcoholic intestinal injury (Chang et al., 2013). In a different study, the probiotic strain *Escherichia coli* Nissle 1917 was shown to up-regulate the expression of the junction-associated protein zonula occludens 1 (ZO-1) in the intestinal epithelial cells of DSS-treated mice (Ukena et al., 2007). Furthermore, some strains of *Lactiplatibacillus plantarum* were shown to enhance intestinal barrier function according to *in vitro* assays based on transepithelial electrical resistance (TEER) measurement (Anderson et al., 2010a) and tight junction genes expression analysis (Anderson et al., 2010b) in Caco-2 epithelial cell layers. Notably, the administration of the probiotic strain *L. paracasei* DG, which is included in the AApf, was recently shown *in vivo* to reduce in mouse colonic mucosa the expression of zonulin (Taverniti et al., 2021), an enzyme that regulates intestinal barrier function promoting intestinal permeability (Fasano, 2011). In our study, the AApf was shown to significantly increase the barrier function in Caco-2 cell layer after 24-h co-incubation, suggesting its potential capability of preventing or reducing intestinal permeability. The mechanisms behind the effects of the AApf or other probiotics on the intestinal epithelial permeability are not precisely known, but it was proposed that the probiotic ability to reduce inflammation at epithelial level can provide a substantial contribution. This hypothesis can be applied in our study, considering our data that showed the ability of the AApf to prevent the activation of the pro-inflammatory transcriptional regulator NF-κB in the Caco-2 intestinal epithelial cell model.

High intensity physical activity increases the production of ROS, most of which are generated in the form of radical superoxide (O_2_^•–^). Experimental evidence showed that ROS, which are associated with various gastrointestinal inflammatory and metabolic disorders (Bhattacharyya et al., 2014), can be detoxified with the contribution of probiotic microorganisms (Wang et al., 2017b). Reportedly, in fact, probiotics can exert significant antioxidant abilities (Lin and Yen, 1999; Persichetti et al., 2014; Wang et al., 2017a). For instance, a high antioxidative effect was reported for *Lacticaseibacillus rhamnosus* and *Lactiplantibacillus plantarum* in ABAP-damaged HepG2 and Caco-2 cell lines, respectively (Wolfe and Liu, 2007; Mu et al., 2019). We found a similar result with the AApf, which significantly reduced ROS accumulation in the Caco-2 cell layer in a dose-dependent fashion also in the presence of different concentrations of the peroxyl radical generator ABAP. Zhang et al. reported that the ability of *Lactiplantibacillus plantarum* C88 to protect Caco-2 cells against oxidative injury was determined by an exopolysaccharide (EPS), which was shown to raise the SOD activity in a dose-dependent manner (Zhang et al., 2013). Interestingly, strain *L. paracasei* DG, which is contained in the AApf, secrete a rhamnose-rich hetero-EPS that covers the outer cell surface (Balzaretti et al., 2017). We can therefore speculate that, similarly to strain C88, the EPS of strain DG could contribute to the antioxidant properties of AApf.

In the intestinal mucosa, oxidative stress and inflammation are interconnected, and contribute to epithelial barrier damage (Chen et al., 2021). Potentially, the AApf ability of reducing ROS in enterocytes, together with the observed anti-inflammatory activity and epithelial barriers preservation, could contribute to protect the integrity of the intestinal mucosa under the stress conditions generated by the strenuous physical activity. Inflammation and oxidative stress are the underlying mechanism of exercise-induced muscle damage, resulting in reduced muscle strength, range of motion and increased muscle soreness. The extent of the muscle damage depends on the intensity and duration of the exercise. While the exercise-induced inflammatory response is crucial to muscle repair and regeneration, nutritional strategies to manage inflammation and oxidative stress are crucial for optimal recovery. Faster recovery allows athletes to train harder and more frequently and results in faster training adaptations and increased performance (Jäger et al., 2016).

This study has several limitations, primarily consisting in the fact that only *in vitro* experiments have been performed, and that the conditions in the gut during physical exercise were only marginally mimicked in our tests. Nonetheless, we used *in vitro* models that are widely adopted in scientific studies and have been demonstrated to be good predictors of the *in vivo* activity. Furthermore, in our opinion, the possibility that the results of this study may effectively prognosticate an effect on human health *in vivo* is favored by two additional facts: 1) we used in the experiments the same industrial formulation available on the market, which has been already employed in a clinical trial (Jäger et al., 2020), and 2) the two bacterial strains in AApf have been already demonstrated *in vivo* to survive the gastrointestinal transit (Balzaretti et al., 2015; Arioli et al., 2018; Radicioni et al., 2019; Koirala et al., 2020).

## Conclusion

This study suggests that the AminoAlta™ probiotic formulation may provide a range of benefits to counteract the gastro-intestinal and immune detrimental consequences of intense physical activity. Specifically, our study suggests that the AApf may exert the activities that are considered the mechanistic bases for the beneficial effects observed in athletes upon probiotic supplementation, such as the prevention of inflammation at the epithelial level, the stimulation of macrophage activity, the preservation of epithelial barrier integrity, and the enhancement of endogenous epithelial oxidant scavenging capacity.

Although derived from *in vitro* experiments, the results of this study provide the rationale for further investigations that will be based on clinical trials aimed to confirm the health-promoting properties of the AminoAlta™ probiotic product in an athletic population.

## Data Availability

The raw data supporting the conclusion of this article will be made available by the authors, without undue reservation.
